# Editorial: Culture and second language (L2) learning in migrants, volume II

**DOI:** 10.3389/fpsyg.2025.1586832

**Published:** 2025-03-21

**Authors:** Adrian Pasquarella, Fanli Jia, Aline Ferreira, John W. Schwieter

**Affiliations:** ^1^School of Education, University of Delaware, Newark, DE, United States; ^2^Department of Psychology, Seton Hall University, South Orange, NJ, United States; ^3^Department of Spanish and Portuguese, University of California, Santa Barbara, Santa Barbara, CA, United States; ^4^Department of Languages and Literatures, Wilfrid Laurier University, Waterloo, ON, Canada

**Keywords:** second language (L2) acquisition, second language learning (L2 learning), migration, social identity, cultural adaptation, acculturation, educational outcomes, multilingual learners

Language acquisition and cultural adaptation are fundamental aspects of migration, shaping individuals' identities, opportunities, and wellbeing. As societies become increasingly multilingual and diverse, understanding the complex interactions between language learning, cultural integration, and social dynamics is more important than ever. Learning a second language (L2) is more than just acquiring words and grammar; it is a deeply personal, social, and cultural experience. This Research Topic brings together cutting-edge research that examines these themes from multiple perspectives, shedding light on L2 learning, heritage language maintenance, migration experiences, and linguistic adaptation.

The studies featured in this Research Topic offer insights into how migrants, refugees, heritage speakers, and international students navigate language learning in different cultural and sociopolitical contexts. Collectively, these contributions deepen our understanding of the relationships between language proficiency, acculturation, social identity, and educational success. Acculturation is a crucial factor in second language acquisition and literacy development among immigrant youth. Our research on Chinese immigrant adolescents in Canada has demonstrated that mainstream acculturation plays a significant role in English literacy development, beyond traditional cognitive factors like vocabulary and reading comprehension (Jia et al., 2014). We have also found that as students strengthen their language skills, their engagement with the broader cultural community increases, creating a reciprocal loop between language proficiency and social integration (Jia et al., 2016). Similarly, Ferreira et al. (2016) found that socio-economic status, language dominance, and heritage affiliation influence reading comprehension in Spanish-English bilinguals, highlighting the sociocultural dimensions of language learning. Additionally, research on Chinese-English bilinguals demonstrates that immigration status and length of exposure to an L2 environment affect reading strategies. Recent immigrants rely more on vocabulary knowledge for reading in both languages, whereas long-term immigrants increasingly depend on phonological awareness in English (e.g., Gottardo et al., 2017).

The first volume of this Research Topic (Pasquarella et al., 2022) explored foundational aspects of second language acquisition, migration, and cultural adaptation, emphasizing cognitive, social, and emotional dimensions. It highlighted the role of identity, linguistic self-perceptions, and educational interventions in shaping language learning experiences. Building on these insights, this second volume further examines how language learning, migration, and cultural adaptation intersect in various multilingual contexts. The articles offer a diverse perspective on the experiences of language learners, heritage speakers, and migrants, examining the psychological, social, and educational factors that shape their linguistic trajectories. Below, we summarize the key contributions of each article in this topic.

Jasemi and Gottardo explored the similarities and differences in second language learning and acculturation between immigrants and refugees in Canada. Their study, focused on Iranian newcomers, highlights that while both groups face language learning challenges, refugees tend to have lower English proficiency due to socioeconomic disadvantages and traumatic experiences. Word reading and vocabulary predicted reading comprehension for immigrants, while only word reading was significant for refugees. Acculturation was positively linked to reading comprehension, and enculturation was negatively associated with vocabulary and reading comprehension for refugees but not for immigrants. These findings underscore the need for tailored language programs that address the distinct linguistic and psychological needs of immigrant and refugee populations.

Buttiler et al. investigated how parental acculturation influences home language practices and children's bilingual development in Chinese American and Mexican American families. Their study revealed that home language input mediates the relationship between parents' cultural orientations and children's heritage language vocabulary. This research highlights the importance of supporting bilingual development through culturally responsive educational policies that recognize the role of parental acculturation in shaping children's linguistic trajectories.

Wen examined the motivational factors driving Chinese heritage language learners to continue studying their ancestral language. Using a mixed-methods approach, the study identifies the “Ideal L2 Self” as a primary predictor of learning effort, with sociocultural contexts playing a crucial role in shaping learners' self-identity. The findings contribute to our understanding of language learning motivation, emphasizing the dynamic interplay between personal identity and cultural heritage in sustaining language proficiency.

Tekin and Trofimovich explored how local residents in Montreal perceive and interact with international students attending English-medium universities. Their study reveals that while both student and non-student locals generally hold positive attitudes, linguistic threat—concerns about the influence of English on French—remains a point of tension. They also find that quality of contact, rather than frequency, is the strongest predictor of positive attitudes. These insights inform strategies for fostering more inclusive and supportive environments for international students in multilingual societies.

Le et al. examine how heritage speakers of Vietnamese in Canada perceive their own cultural belonging and language abilities. The study found that heritage speakers often underestimate how favorably they are perceived by others, which affects their willingness to engage in future interactions. The findings underscore the importance of fostering positive linguistic self-perceptions in heritage language speakers to encourage continued use and transmission of the language.

Ping and Tao introduce an advanced pronunciation training system that integrates multi-sensor detection and algorithmic feedback. Their results show that this technology significantly improves pronunciation accuracy and fluency in English L2 learners compared to traditional methods. This research highlights the potential of technology-enhanced language learning for improving pronunciation instruction in diverse learning contexts.

The studies in this Research Topic collectively advance our understanding of the interplay between language acquisition, cultural adaptation, and identity development in diverse migration contexts. From examining the impact of acculturation on literacy skills to exploring technological advancements in L2 instruction, this collection highlights the dynamic and multidimensional nature of language acquisition.

As global migration continues to shape linguistic landscapes, future research should further explore how sociopolitical factors, educational policies, instructional methods, and technological innovations can support linguistic inclusion and equity. As previous research has shown, language learning is deeply embedded in sociocultural experiences. The findings presented here reinforce the idea that language proficiency and cultural adaptation are mutually reinforcing processes, shaping individuals' identities and social trajectories. We hope that this Research Topic inspires further dialogue and research in multilingualism, multilingual education, and migration studies, ultimately contributing to more equitable and effective language learning environments.
